# Previous exposure to antipsychotic drug treatment is an effective predictor of metabolic disturbances experienced with current antipsychotic drug treatments

**DOI:** 10.1186/s12888-022-03853-y

**Published:** 2022-03-21

**Authors:** Ye Yang, Peng Xie, Yujun Long, Jing Huang, Jingmei Xiao, Jingping Zhao, Weihua Yue, Renrong Wu

**Affiliations:** 1 Department of Psychiatry, and National Clinical Research Center for Mental Disorders, The Second Xiangya Hospital of Central South University, Changsha, 410011 Hunan China; 2 Peking University Sixth Hospital/Institute of Mental Health, Beijing, China

**Keywords:** Antipsychotic, Metabolic side effects, Schizophrenia, Side effects, Weight

## Abstract

**Background:**

Antipsychotic drugs are associated with adverse events, but serious side effects are not frequent. This study aimed to ascertain whether previous exposure to antipsychotic treatment was associated with metabolic disturbances induced by current antipsychotic medication.

**Methods:**

A total of 115 antipsychotic-naïve patients, 65 patients with previous exposure to low-metabolic-risk antipsychotics, and 88 patients with previous exposure to high-metabolic-risk antipsychotics were enrolled in our case-control study. All patients were administered olanzapine. Body weight, body mass index (BMI), biochemical indicators of blood glucose and lipids, the proportion of patients who gained more than 7% of their body weight at baseline, and the percentage of dyslipidemia were evaluated. All assessments were conducted at baseline and at 4 and 6 weeks after treatment.

**Results:**

Olanzapine treatment resulted in a significant increase in body weight and BMI in antipsychotic-naïve patients compared with the other two groups (both *p* < 0.05). However, increases in lipid levels in the high-metabolic-risk antipsychotics group were significantly higher than that in the other two groups (both *p* < 0.05). A history of antipsychotics use was not associated with weight gain (all *p* > 0.05). Higher low-density lipoprotein cholesterol ≥3.37 mmol/L^–1^ was observed in antipsychotics exposure group compared with no history of antipsychotics exposure (aOR, 1.75; 95% CI, 1.07-3.52). Particularly, a history of high-metabolic-risk antipsychotics use was associated with a higher risk of LDL-C ≥3.37 mmol/L–1(aOR, 2.18; 95% CI, 1.03-3.32) compare with other two groups.

**Conclusions:**

A history of exposure to antipsychotics, particularly high-metabolic-risk antipsychotics, is associated with current antipsychotic-induced metabolic disturbances.

**Supplementary Information:**

The online version contains supplementary material available at 10.1186/s12888-022-03853-y.

## Background

Schizophrenia is a severe mental disease with an estimated lifetime morbidity risk approaching 1% worldwide and an associated 15- to 20-year reduction in life expectancy compared to the general population [1]. The use of atypical antipsychotics (AAPs) is associated with frequent occurrence of metabolic side effects, including weight gain, dyslipidemia, and glucose intolerance [2]. The prevalence of metabolic abnormalities in patients previously treated with antipsychotics is 35.3%, which is significantly higher than that in first-episode patients (9.9%) and patients not using antipsychotics (9.8%), suggesting that antipsychotics play a vital role in the high prevalence of metabolic disorders in patients with schizophrenia [3].

Weight gain and metabolic abnormalities are reliable predictors of cardiovascular morbidity and are subsequently related to poorer quality of life, increased risk of nonadherence, and reduced life expectancy. However, recent studies have reported that long-term antipsychotic treatment decreases mortality rates and hospitalizations in patients with schizophrenia [4]. Understanding the metabolic risks of antipsychotics and methods of monitoring and predicting these risks are essential parts of psychiatric practice. Regular monitoring of metabolic measurements before and during antipsychotic therapy is recommended as secondary prevention [5]. However, it has been reported that patients with severe mental illness do not undergo appropriate metabolic screening because of lack of time, lack of resources, time incongruities, and patient noncompliance [6, 7]. Physiological and demographic factors that predict metabolic dysregulation associated with antipsychotics also have vital roles in metabolic monitoring. Sex, age, smoking, weight at baseline, and early changes in fasting triglycerides, glucose, and weight are predictive of long-term changes in metabolic measurements during treatment [8–10]; genetic vulnerability also has an appreciable impact on antipsychotic induced adverse drug reactions [6]. However, the validity of these predictors has been disputed because different studies have reported heterogeneous results.

Another possible predictor is the history of antipsychotics use. Regardless of whether the history of antipsychotics use is long-term or short-term it is accompanied by metabolic abnormalities. However, it is not clear whether previous exposure to antipsychotics can affect the metabolic changes caused by current antipsychotic medications. A new concept called “metabolic memory” in the field of diabetes has proposed that gestational diabetes is responsible for the onset of diseases, such as type 2 diabetes and obesity associated with metabolic syndrome, in offspring during adulthood [11]. Therefore, it has been speculated that patients with schizophrenia exhibit metabolic memory after exposure to antipsychotics. Additionally, weight gain was more pronounced with short-term treatment than with long-term treatment [12]; therefore, it remains unknown whether the duration of previous antipsychotic treatment can exert different effects on the metabolic disturbances induced by current antipsychotic treatment. AAPs differ markedly in their potential to cause metabolic disturbances. Clozapine and olanzapine are associated with the highest risk of clinically significant metabolic disturbances, followed by risperidone and quetiapine; however, ziprasidone and aripiprazole are associated with minimal metabolic risk [13, 14]. Whether patients with schizophrenia and different histories of exposure to antipsychotics with different metabolic risks experience different metabolic risks with their current antipsychotic drug treatment remains unclear.

Although antipsychotics are widely used for the treatment of psychiatric conditions, the monitoring and screening of patients is inadequate [6]. This study aimed to explore the metabolic risks of patients with different histories of antipsychotic drug usage. This study may provide a novel and convenient method of predicting the metabolic risk of antipsychotic treatment, thereby leading to early interventions for metabolic syndrome and prevention of long-term negative outcomes.

## Methods

### Participants

All procedures were approved by the institutional review boards of the Second Xiangya Hospital of Central South University and Peking University Sixth Hospital. The study was conducted between December 2019 and April 2021, and all subjects provided written informed consent in accordance with the Declaration of Helsinki. Participants met the diagnostic criteria for schizophrenia based on the *Diagnostic and Statistical Manual of Mental Disorders* fifth edition. Two-hundred-and-seventy inpatients and outpatients, 18 to 55 years of age, including antipsychotic-naïve/first-episode patients and chronic patients, were recruited for the study. The antipsychotic-naïve/first-episode patients had not been exposed to antipsychotics before enrollment in our study and chronic patients whose condition was stable and who had discontinued medication for at least 3 months according to doctor’s advice were enrolled. Antipsychotics included AAPs such as olanzapine, clozapine, risperidone, aripiprazole, quetiapine; and typical antipsychotics, such as haloperidol, amisulpiride, and chlorpromazine. Antipsychotic drugs start with an initial low dose which is gradually increased to the therapeutic dose. During the treatment period, the drug dose is adjusted according to the needs of the patient. Patients with a history of antipsychotics use receive medication regularly under the supervision of a guardian for most of the duration of their disease; therefore, the duration of treatment was proportional to the course of the disease. Because it was impossible to accurately calculate the previous antipsychotics treatment duration, we estimated the course of the disease. Antipsychotics mentioned in the current study are classified as having a high, medium, or low risk of inducing metabolic abnormalities according to previous literature [15], and because fewer patients were using low-metabolic-risk drugs, we classified both intermediate- and low-metabolic-risk antipsychotics into the low-metabolic-risk group. The high-metabolic-risk antipsychotics included clozapine and olanzapine and the low-metabolic-risk antipsychotics were risperidone, quetiapine, ziprasidone, aripiprazole, amisulpiride, and haloperidol. All participants enrolled in our study were treated with olanzapine (15–20 mg/day at 8:00 pm) for 6 weeks. The initial dose of olanzapine was 5 mg/day, which was then gradually adjusted to 15–20 mg/day according to the patients’ condition. The use of trihexyphenidyl for extrapyramidal symptoms or benzodiazepines for insomnia or agitation was allowed, if necessary.

The exclusion criteria were as follows: clinical medical abnormalities, including nervous system diseases, major physical illnesses, or significant medical illnesses; disorders such as intellectual disability, alcohol or substance abuse, and other specific systemic diseases; a history of eating disorders; previous treatment with polypharmacy that included both high and low metabolic risk antipsychotics; strict diet within a month of screening or during the study; pregnancy or breastfeeding; need for different drugs because of patient condition; and inability to adhere to treatment.

### Data collection

Data was collected at the time of enrollment. Baseline data included demographic information, comprehensive medical history, clinically evaluated psychiatric symptoms, physical examination results (including weight and height), and laboratory test results (including fasting low-density lipoprotein cholesterol [LDL-C], triglycerides, cholesterol, high-density lipoprotein cholesterol [HDL-C], glucose levels, and liver and renal function test results). Weight measurements were performed at 8:00 am after overnight fasting. The body mass index (BMI) was calculated as body weight/height (kg/m^2^). Clinical follow-up was performed 4 and 6 weeks after treatment. During each follow-up visit, all evaluations (physical examinations, laboratory tests, weight measurements, clinical symptom evaluations, and assessments of adverse effects) were repeated. The primary outcomes included changes in weight; BMI; fasting glucose; and lipid profiles, including triglycerides, cholesterol, HDL-C, and LDL-C. The secondary outcome was the proportion of patients who gained more than 7% of their baseline body weight after 6 weeks. Dyslipidemia was defined as cholesterol ≥5.18 mmol/L^-1^, triglycerides ≥1.70 mmol/L^-1^, HDL-C <1.04 mmol/L^-1^, or LDL-C ≥3.37 mmol/L^-1^, based on the Chinese guidelines for dyslipidemia [16].

### Statistical analysis

The Statistical Package for Social Sciences (version 25.0) was used for statistical analyses. Since our data did not follow a normal distribution, the 2-tailed Mann–Whitney U or Kruskal–Wallis tests were used to evaluate between-group differences in changes in body weight, BMI, fasting glucose, triglycerides, cholesterol, HDL-C, and LDL-C from baseline to each time point. The chi-square or Fisher’s exact tests were used to test differences in the distribution of categorical variables. Further comparisons between groups were carried out using the Dunn–Bonferroni post-hoc test. Continuous variables were described using means and standard deviations. Categorical variables were described using frequencies and percentages. Logistic regression models were used to assess the adjusted effect of a history of antipsychotics use on different metabolic risks for olanzapine-induced weight gain and metabolic disturbances in patients with schizophrenia (compared to previously untreated patients). Statistical significance was set at *P* < 0.05.

## Results

**Fig. 1:**
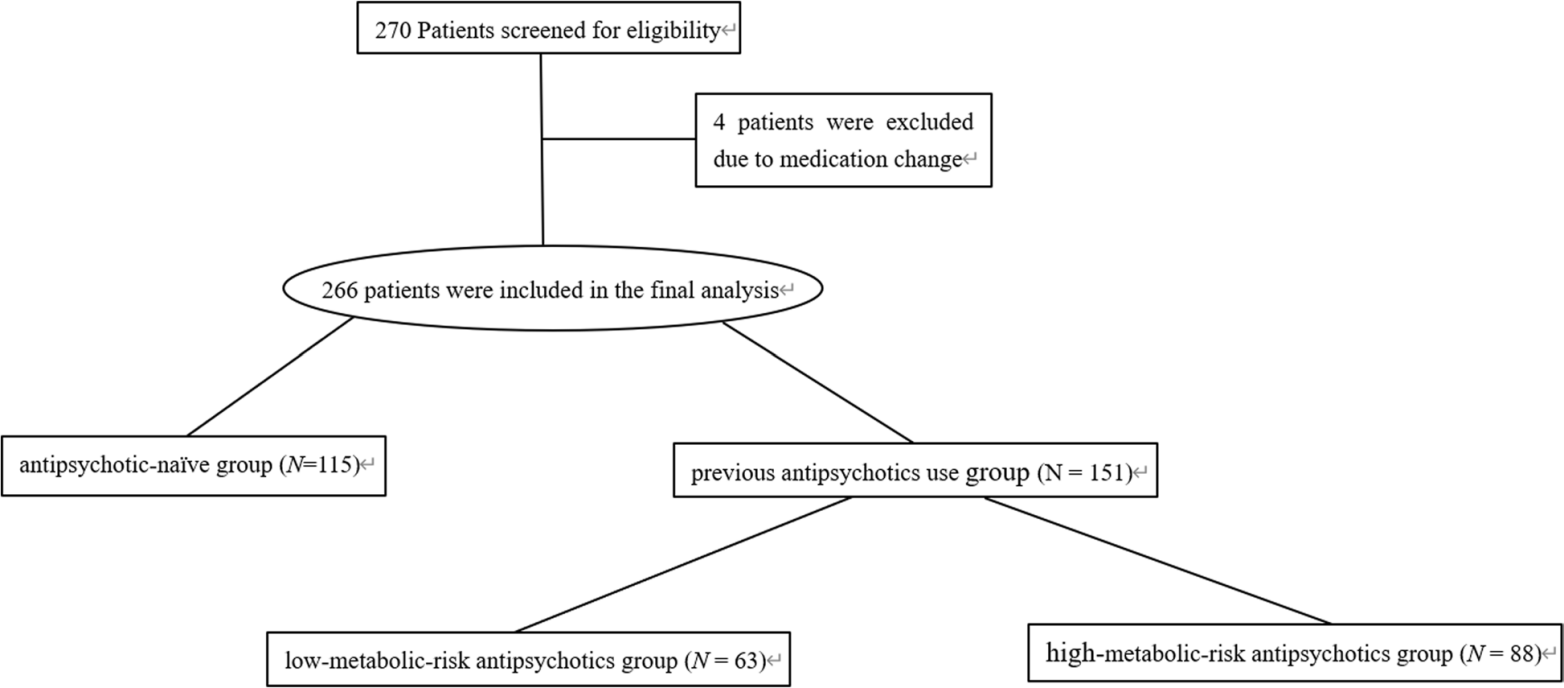
Flowchart of participation in the study

A total of 270 schizophrenia inpatients and outpatients (mean age, 29.8 years; range, 18–48 years) were enrolled in the study. Four patients were excluded because they were unable to tolerate olanzapine treatment; therefore, 266 patients were included in the final analysis (Fig. 1). The proportions of female and male patients were similar (51.1% and 48.9%; 136 and 130, respectively). The mean duration of schizophrenia was 6.3 ± 5.3 years (range, 0.5–22.5 years). All patients had a BMI in the normal range at baseline (mean BMI, 22.5 ± 4.5 kg/m^2^).

### Changes in body weight, BMI, and metabolic disturbance after olanzapine treatment

Significant increases in weight and BMI were observed after olanzapine treatment (Table 1). The mean weight gain was 3.1 kg and 2.1 kg after 6 and 4 weeks of olanzapine treatment, respectively. Furthermore, 31.2% (83/266) of all patients had increased their initial body weight by more than 7% after 6 weeks of olanzapine treatment. At 6 weeks, 27.8% (74/266) of the patients were overweight (BMI >25 kg/m^2^). Significant increases in triglyceride levels, cholesterol levels, and LDL-C levels were observed at 6 weeks (Table 1). No dyslipidemia was observed in patients at baseline; however, 70.3% (187/266) of patients had dyslipidemia after 6 weeks of olanzapine treatment, based on the Chinese guidelines for dyslipidemia.

**Table 1:** Changes in weight and metabolic parameters after olanzapine treatment

Variable and Week	*Mean*	*SD*	*P*
Weight (kg)			
Weight 0	60.06	10.96	
Weight 4	62.28	10.86	<0.001
Weight 6	63.16	10.91	<0.001
Body mass index (kg/m^2^)			
Weight 0	22.53	4.57	
Weight 4	23.42	4.71	<0.001
Weight 6	23.75	4.71	<0.001
Triglycerides (mmol l^−1^)			
Weight 0	1.22	0.71	
Weight 4	1.85	1.20	<0.001
Weight 6	1.84	1.12	<0.001
Cholesterol (mmol l^−1^)			
Weight 0	4.21	0.89	
Weight 4	4.56	0.94	<0.001
Weight 6	4.56	0.91	<0.001
HDL-C (mmol l^−1^)			
Weight 0	1.34	0.38	
Weight 4	1.31	0.35	0.171
Weight 6	1.32	0.38	0.621
LDL-C (mmol l^−1^)			
Weight 0	2.41	0.76	
Weight 4	2.65	0.85	<0.001
Weight 6	2.62	0.84	<0.001
Fasting glucose (mmol l^−1^)			
Weight 0	4.87	0.67	
Weight 4	4.86	0.87	0.894
Weight 6	4.93	0.80	0.113

*HDL-C* high-density lipoprotein cholesterol, *LDL-C* low-density lipoprotein cholesterol

### Weight increase and olanzapine-induced metabolic disturbance in the antipsychotic-naïve group and the groups with a history of antipsychotics exposure

To detect the effect of a history of antipsychotics exposure on olanzapine-induced weight gain and metabolic disturbance, we classified the patients into two groups: the antipsychotic-naïve group (*N*=115) and the previous antipsychotics use group (*N* = 151). Significant differences in the increases in body weight and LDL-C were observed between the two groups (Table 2). Interestingly, their rates of increase showed opposite trends: the weight of the antipsychotic-naïve group increased by 2.8 kg (week 4) and 4.2 kg (week 6) from baseline (*F* = 3.29;* p* < 0.001), while that of the previous antipsychotics use group increased by only 1.6 kg (week 4) and 2.2 kg (week 6) (*Z* = 4.53; *p* < 0.001) during olanzapine treatment. Similarly, the antipsychotic-naïve group was more likely to have an increased BMI (*Z* =2.83; *p* = 0.005), with increases of 0.7% (week 4) and 1.8% (week 6) compared with 0.3% (week 4) and 1.3% (week 6) in the previous antipsychotics use group (*Z* = 4.31; *p* < 0.001). In contrast, LDL-C exhibited a higher rate of increase in the previous antipsychotics use group (*Z* = -2.19;* p* =0.030) than in the antipsychotic-naïve group (*F* = -3.89; *p* < 0.001).

**Table 2:** Comparison of changes in body weight and metabolic parameters over time, between the antipsychotic-naïve and previous antipsychotic-use groups

Variable increase	Antipsychotic-naïve group	Previous antipsychotic-use group	*Z*	*P*	*ES*
Weight (kg)					
Week 4	2.84±3.35	1.59±2.49	3.29	<0.001	0.297
Week 6	4.24±3.90	2.22±3.35	4.53	<0.001	0.296
BMI (kg/m^2^)					
Week 4	0.73±1.76	0.28±0.67	2.83	0.005	0.298
Week 6	1.77±2.37	1.30±0.88	4.31	<0.001	0.296
Cholesterol (mmol l^−1^)					
Week 4	0.15±1.35	0.31±1.19	-0.96	0.338	0.302
Week 6	0.45±0.91	0.35±0.92	0.86	0.391	0.300
Triglyceride (mmol l^−1^)					
Week 4	0.59±1.08	0.71±0.92	0.02	0.984	0.301
Week 6	0.68±0.65	0.61±1.10	0.70	0.486	0.300
HDL-C (mmol l^−1^)					
Week 4	-0.11±0.44	-0.04±0.55	-0.90	0.369	0.302
Week 6	0.00±0.41	-0.03±0.33	0.26	0.435	0.301
LDL-C (mmol l^−1^)					
Week 4	0.06 ±0.85	0.30±0.81	-2.19	0.030	0.303
Week 6	0.23±0.67	0.56±0.75	-3.89	<0.001	0.306
Fasting glucose (mmol l^−1^)					
Week 4	-0.13±1.06	0.00±1.03	-0.92	0.358	0.302
Week 6	0.20±0.74	0.04±0.81	1.67	0.096	0.299

*HDL-C* high-density lipoprotein cholesterol, *LDL-C* low-density lipoprotein cholesterol

### Weight increase and olanzapine-induced metabolic disturbance in the antipsychotic-naïve group and groups with exposure to antipsychotics with different metabolic risks

To compare the effect of the use of antipsychotics with different metabolic risks on olanzapine-induced weight gain and metabolic disturbances, we further classified the patients into three groups (antipsychotic-naïve group, *N* = 115; low-metabolic-risk antipsychotics group, *N* = 63; and high-metabolic-risk antipsychotics group, *N* = 88). There were significant differences in the increases in body weight and LDL-C among the different groups at 6 weeks (both *p* < 0.001) (Table 3). The post hoc analysis revealed that weight gain in the high-metabolic-risk antipsychotics group was significantly lower than that in the antipsychotic-naïve and low-metabolic-risk antipsychotics groups (both *p* < 0.001); however, this remarkable difference was not observed between the antipsychotic-naïve group and low-metabolic-risk antipsychotics group (*p* = 0.187). In contrast, the increase in LDL-C in the high-metabolic-risk antipsychotics group was significantly higher than that in the other two groups (both *p* < 0.001), and no significant difference was observed between the antipsychotic-naïve and low-metabolic-risk antipsychotics groups (*p* = 0.690).

**Table 3:** Comparison of changes in body weight and metabolic parameters over time between the antipsychotic-naïve, low-metabolic risk antipsychotics, and high-metabolic risk antipsychotics groups

Variable	Antipsychotic-naïve group	Low-metabolic risk antipsychotics group	High-metabolic risk antipsychotics group	*H*	*P*	*ES*
Weight (kg)						
Week 4	2.84±3.35	2.53±1.86	0.92±2.67	11.87	<0.001	0.358
Week 6	4.24±3.90	3.51±2.16	1.30±3.74	18.07	<0.001	0.352
BMI (kg/m^2^)						
Week 4	0.73±1.76	0.53±0.55	0.10±0.69	6.43	0.002	0.363
Week 6	1.77±2.37	1.30±0.88	0.44±1.33	13.79	<0.001	0.356
Cholesterol (mmol l^−1^)						
Week 4	0.15±1.35	0.35±1.17	0.29±1.21	0.501	0.607	0.369
Week 6	0.45±0.91	0.23±0.83	0.44±0.98	1.28	0.281	0.368
Triglycerides (mmol l^−1^)						
Week 4	0.59±1.08	0.71±0.92	0.51±0.97	0.700	0.498	0.369
Week 6	0.68±0.65	0.61±1.10	0.57±1.20	0.260	0.768	0.369
HDL-C (mmol l^−1^)						
Week 4	-0.11±0.44	0.01±0.44	-0.08±0.62	0.815	0.444	0.368
Week 6	0.00±0.41	-0.01±0.33	-0.04±0.33	0.410	0.663	0.369
LDL-C (mmol l^−1^)						
Week 4	0.57±0.85	0.28±0.83	0.31±0.81	2.39	0.094	0.367
Week 6	0.23±0.67	0.27±0.65	0.80±0.74	18.43	<0.001	0.352
Fasting glucose (mmol l^−1^)						
Week 4	-0.13±1.06	0.01±0.91	-0.01±1.11	0.43	0.65	0.369
Week 6	0.20±0.74	0.14±0.76	-0.03±0.85	2.27	0.106	0.367

*HDL-C* high-density lipoprotein cholesterol, *LDL-C* low-density lipoprotein cholesterol

### Treatment duration stratification analysis

**Fig. 2:**
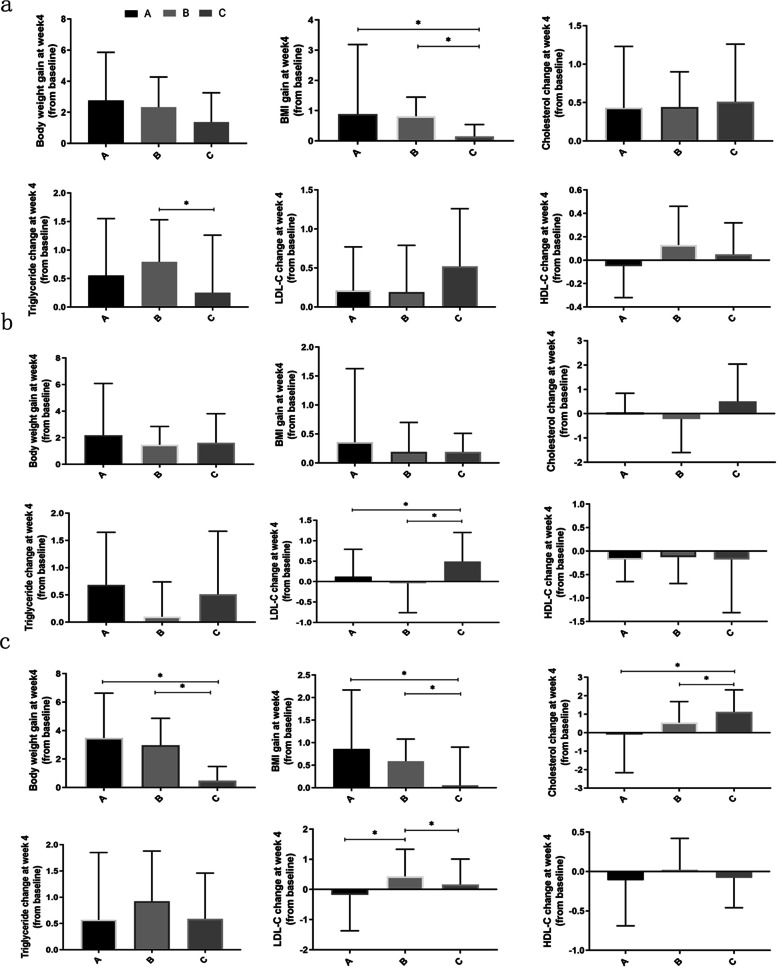
Subgroup analysis of changes in body weight and metabolic parameters among different groups after 4 weeks of olanzapine treatment. **a** Changes in body weight and metabolic parameters among different groups with 1 to 2 years of previous antipsychotics exposure. **b** Changes in body weight and metabolic parameters among different groups with 2 to 5 years of previous antipsychotics exposure. **c** Changes in body weight and metabolic parameters among different groups with more than 5 years of previous antipsychotics exposure. **A**, **B**, and **C** represent the antipsychotic-naïve group, low-metabolic-risk antipsychotics group, and high-metabolic-risk antipsychotics group, respectively

**Fig. 3:**
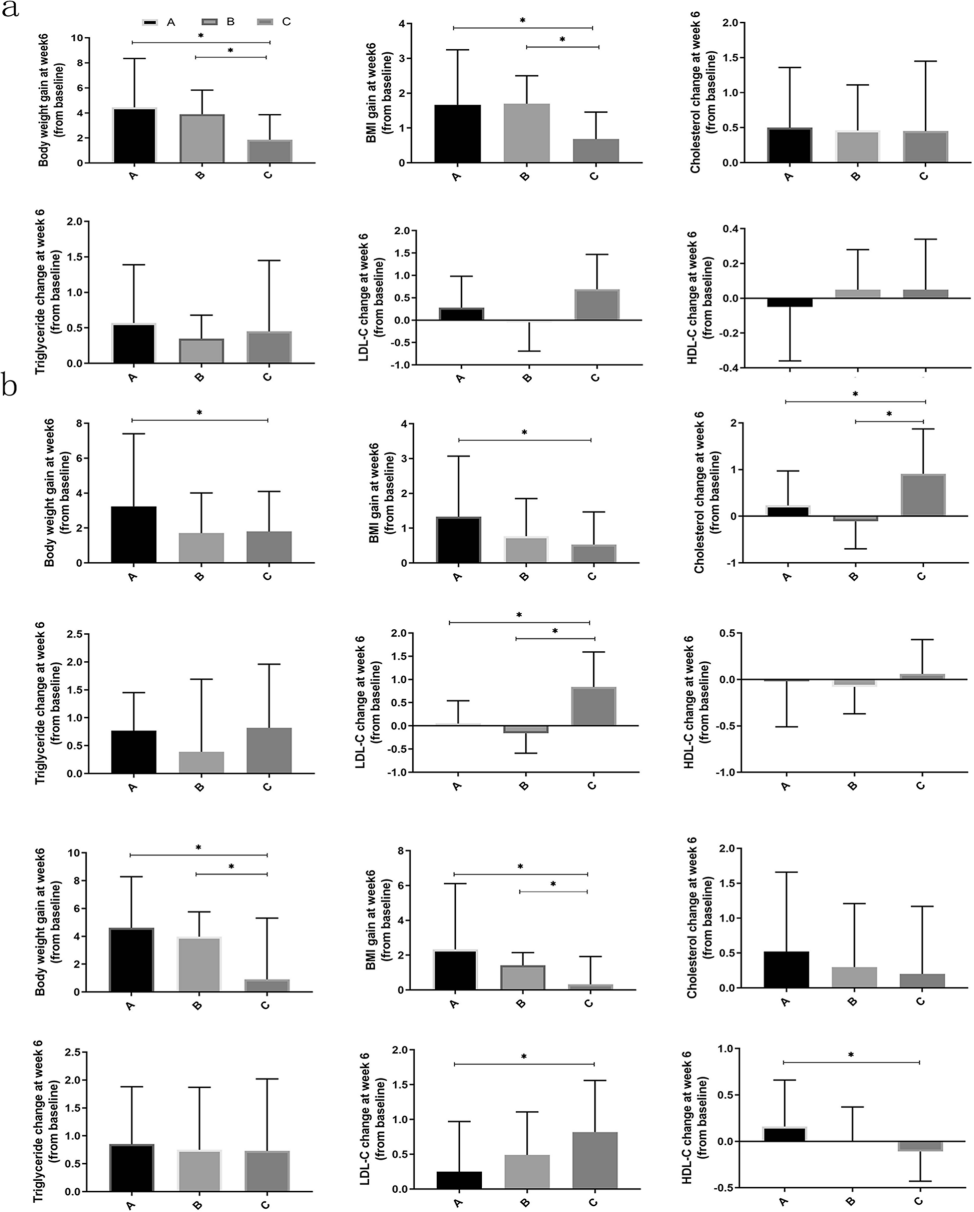
Subgroup analysis of changes in body weight and metabolic parameters among different groups after 6 weeks of olanzapine treatment. **a** Changes in body weight and metabolic parameters among different groups with 1 to 2 years of previous antipsychotics exposure. **b** Changes in body weight and metabolic parameters among different groups with 2 to 5 years of previous antipsychotics exposure. **c** Changes in body weight and metabolic parameters among different groups with more than 5 years of previous antipsychotics exposure. **A**, **B**, and **C** represent the antipsychotic-naïve group, low-metabolic-risk antipsychotics group, and high-metabolic-risk antipsychotics group, respectively

Because of the unbalanced distribution of disease duration, stratification analyses were conducted according to previous treatment durations (Figs. 2 and 3). We divided duration into less than 2 years, 2 to 5 years, and more than 5 years; then, we further explored the effects of antipsychotics with different metabolic risks and different durations of antipsychotics exposure on metabolism after olanzapine treatment.

When the treatment duration was within 2 years, a significant difference in the increase in body weight was observed between the previously treated and untreated groups, with a higher increase observed in the antipsychotic-naïve group (*H* = 9.12; *p* = 0.043) (Supplementary Table 1) after 6 weeks of olanzapine treatment. A further subgroup analysis revealed a similar difference in the increase in body weight among different groups after 6 weeks of olanzapine treatment (*H* = 9.12; *p* = 0.010), but not after 4 weeks of olanzapine treatment (*H*= 3.61; *p* = 0.165) (Fig. 2, Fig. 3, and Supplementary Table 2). The increase in BMI in the high-metabolic-risk antipsychotics group was significantly lower than that in the antipsychotic-naïve (*H* = 7.56; *p* = 0.023) and the low-metabolic-risk antipsychotics (*H* = 8.55; *p* = 0.014) groups after 4 and 6 weeks of olanzapine treatment. Regarding metabolic disturbances, we observed a significant increase in fasting glucose in the previous antipsychotics use group after 4 weeks of olanzapine treatment (*H* = -2.49; *p* = 0.016). Further subgroup analysis did not reveal any differences in glycemic changes (Supplementary Figure 1 and Table 2). Unexpectedly, the increase in triglycerides was significantly higher in the low-metabolic-risk antipsychotics group than in the high-metabolic-risk antipsychotics group (*p* = 0.043) after 4 weeks of olanzapine treatment (Fig. 2, Fig. 3, and Supplementary Table 2). However, there were no significant differences in the proportion with body weight gain exceeding 7% of the initial body weight and dyslipidemia among the different groups (all *p* > 0.05) (Supplementary Tables 3 and 4).

When the treatment duration ranged from 2 to 5 years, no significant differences in the increases in body weight and lipids were observed between the previously treated and untreated groups (both *p* > 0.05) (Supplementary Table 1). The increases in weight and BMI were significantly lower in the high-metabolic-risk antipsychotics group (*H* = 2.63; *p* = 0.008) than in the antipsychotic-naïve group (*H* = 2.97; *p* = 0.009) (Fig. 2, Fig. 3, and Supplementary Table 2). In contrast, the increase in cholesterol in the high-metabolic-risk antipsychotics group was significantly higher than that in the antipsychotic-naïve and low-metabolic-risk antipsychotics groups after 6 weeks of olanzapine treatment (both *p* < 0.05). Similarly, the high-metabolic-risk antipsychotics group showed higher increases in LDL-C than did the other groups after 4 weeks and 6 weeks of olanzapine treatment (all *p* < 0.05). The proportions of patients who gained more than 7% of their initial body weight were significantly different among the different groups, with a significantly lower proportion in the high-metabolic-risk antipsychotics group than in the other two groups (both *p* < 0.05) (Supplementary Table 4). Interestingly, an opposite trend in the percentage of dyslipidemia was observed among groups: 36.8% (7/19) and 50% (9/18) in the high-metabolic-risk antipsychotics group had cholesterol ≥5.18 mmol/L^-1^ and LDL-C ≥3.37 mmol/L^-1^ at week 6, respectively; these levels were significantly higher than those in the antipsychotic-naïve and low-metabolic-risk antipsychotics groups (Supplementary Table 3).

When the treatment duration was more than 5 years, significant differences were observed in increases in weight and BMI between the previously treated and untreated groups after olanzapine treatment (all *p* < 0.05), with a significant increase observed in the previously untreated group (Supplementary Table 1). Similarly, significantly higher HDL-C was also observed in the previously untreated group at week 4 (*H* = 2.60; *p* = 0.010). Furthermore, we observed a significant increase in LDL-C in the treated group at weeks 4 and 6 (both *p* < 0.05). A further subgroup analysis revealed significant differences in body weight and BMI among different groups (all *p* < 0.05) (Fig. 1, Fig. 2, and Supplementary Table 2) and indicated a gradient decreasing trend; the increases were higher in the antipsychotic-naïve group than in the low-metabolic-risk antipsychotics group, and they were higher in the low-metabolic-risk antipsychotics group than in the high-metabolic-risk antipsychotics group. Conversely, the increases in blood lipids showed the opposite trend after olanzapine treatment, and the increases in cholesterol and LDL-C showed significant differences among groups after 4 weeks and 6 weeks of olanzapine treatment (all *p* < 0.05), with significantly higher rates of increase in the low-metabolic-risk antipsychotics group and the high-metabolic-risk antipsychotics group compared with the antipsychotic-naïve group. The increase in HDL-C was significantly lower in the low-metabolic-risk antipsychotics group and high-metabolic-risk antipsychotics group compared to the antipsychotic-naïve group (all *p* < 0.05) and 33.3% (13/39) of patients in the low-metabolic-risk antipsychotics group experienced an increase in their initial body weight of more than 7%; this proportion was significantly higher than that in the other groups (Supplementary Table 4). Regarding dyslipidemia, HDL-C <1.04 mmol/L^-1^ mainly occurred in the high-metabolic-risk antipsychotics group (*p* < 0.05) (Supplementary Table 3).

### Effects of exposure to antipsychotics with different metabolic risks on olanzapine-induced weight gain and metabolic disturbances

We used logistic regression models to evaluate the adjusted effects of exposure to antipsychotics with different metabolic risks on the risks of weight gain and metabolic disturbances after olanzapine treatment (compared to those without a history of antipsychotic drug use). Variables previously reported to predict adverse metabolic effects of antipsychotics in multiple studies, such as age, sex, weight at baseline and early changes in weight were included in multivariate conditional logistic regression models. We included weight gain exceeding 7% of the initial body weight, triglycerides ≥1.70 mmol/L^˗1^, cholesterol ≥5.18 mmol/L^–1^, HDL-C <1.04 mmol/L^–1^, and LDL-C ≥3.37 mmol/L^–1^ as dependent variables, and exposure to antipsychotic drugs, age, sex, weight at baseline and early changes in weight as independent variables in the logistic regression analysis. Univariate conditional logistic regression models were used to assess the univariable association of various characteristics with metabolic adverse reactions. Further multivariate conditional logistic regression models were used to evaluate the adjusted effect of different metabolic-risk antipsychotics on the risk of metabolic adverse reactions. The variables related to metabolic adverse reactions screened by univariate logistic regression were included in the multiple logistic regression analysis.

**Fig. 4:**
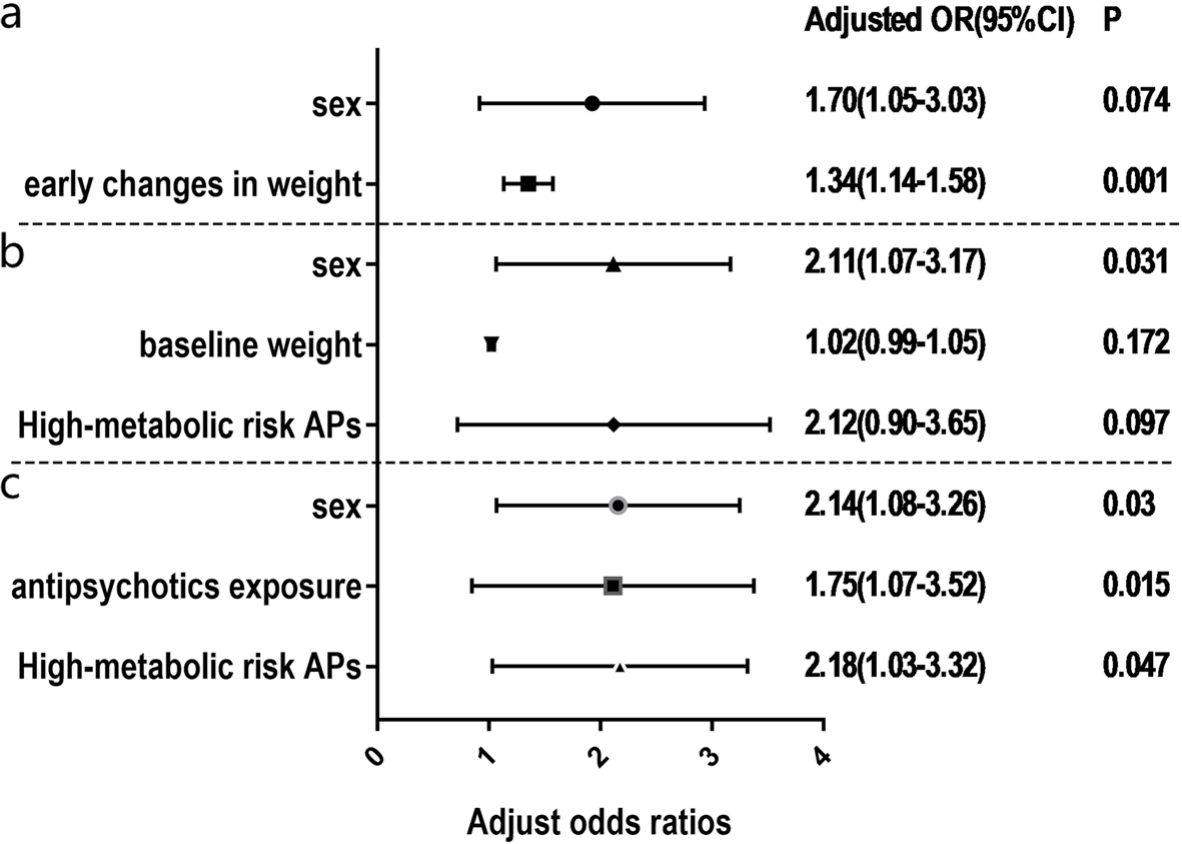
Forest plot of the multivariate analyses for the risk of metabolic adverse reactions. **a** Adjusted odds ratios and two-sided 95% confidence intervals of weight gain of more than 7% of initial weight after previous antipsychotic treatment compared to no previous antipsychotic treatment in different metabolic risk subgroups. **b** Adjusted odds ratios and two-sided 95% confidence intervals of HDL-C <1.04 mmol/L^–1^ after previous antipsychotic treatment compared to no previous antipsychotic treatment in different metabolic risk subgroups. **c** Adjusted odds ratios and two-sided 95% confidence intervals of LDL-C ≥3.37 mmol/L^–1^ after previous antipsychotic treatment compared to no previous antipsychotic treatment in different metabolic risk subgroups. APs, history of exposure to antipsychotics; high-metabolic-risk APs, history of exposure to high-metabolic-risk antipsychotics; low-metabolic-risk APs, history of exposure to low-metabolic-risk antipsychotics

Univariate conditional logistic regression analyses revealed that age was a risk factor for cholesterol ≥5.18 mmol/L–1(aOR, 1.052; 95% CI, 1.015-1.090) (Supplementary Table 5). Sex was a risk factor for weight gain exceeding 7% of the initial body weight (aOR, 1.285; 95% CI, 0.763-2.163), HDL-C <1.04 mmol/L^–1^ (aOR, 2.581; 95% CI, (1.378-3.834), and LDL-C ≥3.37 mmol/L^–1^ (aOR, 2.137; 95% CI, 1.106-3.326). Baseline weight was a risk for triglycerides ≥1.70 mmol/L^˗1^ (aOR, 1.030; 95% CI, 1.007-1.054) and LDL-C ≥3.37 mmol/L^–1^ (aOR, 1.038;95% CI, 1.010-1.066). Early changes in weight were significantly associated with weight gain exceeding 7% (aOR, 1.340; 95% CI, 1.137-1.579). Antipsychotics exposure was a risk for LDL-C ≥3.37 mmol/L–1(aOR, 1.768; 95% CI, 1.005-3.531). High-metabolic-risk antipsychotics exposure was associated with a higher risk of HDL-C <1.04 mmol/L^–1^ (aOR, 2.084; 95% CI, 1.068-4.069) and LDL-C ≥3.37 mmol/L^–1^ (aOR, 2.067; 95% CI, 1.069-4.441). Further multivariate conditional logistic regression models also revealed an association between early changes in weight and weight gain exceeding 7% (aOR, 1.338; 95% CI, 1.135-1.579) (Fig. 4). The association between sex and HDL-C <1.04 mmol/L^–1^ (aOR, 2.111; 95% CI, 1.069-3.169) and LDL-C ≥3.37 mmol/L^–1^(aOR, 2.143; 95% CI, 1.078-3.258) was also observed. Antipsychotics exposure was associated with a 1.7-fold increased risk of LDL-C ≥3.37 mmol/L–1 compared with no history of antipsychotics exposure (aOR, 1.753; 95% CI, 1.072-3.523). Furthermore, a history of high-metabolic-risk antipsychotics use was associated with a higher risk of LDL-C ≥3.37 mmol/L–1(aOR, 2.18; 95% CI, 1.034-3.315).

## Discussion

To the best of our knowledge, this is the first clinical study to explore the effects of a history of antipsychotic drug use with different metabolic risks on olanzapine-induced weight gain and metabolic disturbances in patients with schizophrenia. This study revealed that patients with schizophrenia who used antipsychotics are particularly vulnerable to antipsychotic-induced metabolic abnormalities, with heterogeneity observed with medications with different metabolic risks and treatment durations. However, antipsychotic-naïve patients easily experience weight gain compared with patients treated with previous antipsychotics.

According to this study, first-time antipsychotic drug use was associated with more pronounced weight gain, and patients with a history of antipsychotic drug use easily developed dyslipidemia after olanzapine treatment. It is interesting to note that the increase in body weight was not exactly consistent with the increases in metabolic parameters. Additionally, weight gain was independent of the previous treatment duration, with the highest increase observed in previously unmedicated patients and the lowest increase observed in patients who previously used high-metabolic-risk antipsychotics. This is in accordance with previous reports that indicated that drug-naïve patients gain significantly more weight than patients with substantial previous antipsychotics exposure [17]. Weight gain reaches a plateau after a significant increase during the early stages of treatment, especially with high-metabolic-risk antipsychotics; the majority of weight gain occurs during the first 3 months of treatment [18, 19]. Therefore, after experiencing significant weight gain during the early stages of treatment, weight gain during the later stages will not be significant and may be unaffected by the previous treatment duration. In contrast, the changes in metabolic parameters did not reach a plateau during short-term treatment, which may reasonably explain the differences between weight increases and metabolic parameters. Therefore, our study suggests that even if the weight gain risks of AAPs increase the probability of hyperglycemia and dyslipidemia, weight gain is not exactly consistent with metabolic changes, which is consistent with a previous study [12]. All these results suggest that, in addition to the effects mediated by changes in weight, some antipsychotics may have independent effects on metabolic profiles.

In contrast to weight gain, the increases in blood lipids were more significant in patients with a history of antipsychotic drug use. However, the results of previous studies are quite heterogeneous, and many studies have provided convincing evidence that individuals experiencing their first episode of psychosis could be particularly susceptible to metabolic dysfunction when using AAPs [17], which is not consistent with our results. Although previous results indicated that patients with schizophrenia who had previously used antipsychotics had a higher prevalence of dyslipidemia than patients with schizophrenia who had not previously used antipsychotics, in this study, patients who had previously used antipsychotics had an overall higher prevalence of abnormal lipid profiles, which is consistent with our result indicating that a higher increase in LDL-C was observed in patients with schizophrenia who had previously used antipsychotics [20]. It is worth mentioning that this was a cross-sectional study that did not further distinguish the current antipsychotics of enrolled patients. Our study measured body weight and metabolic parameters after 6 weeks of olanzapine treatment; therefore, our results need further validation.

Further analysis demonstrated that previous exposure to high-metabolic-risk antipsychotics resulted in more significant increases in LDL-C, that intensified with increased duration of previous antipsychotic drug exposure. It is apparent that high-metabolic-risk antipsychotics are associated with the largest degree of metabolic dysregulation, but there is insufficient data to evaluate the effects of previous high-metabolic-risk antipsychotic drug use on metabolic abnormalities induced by the current antipsychotic drug. However, switching antipsychotics to relatively weight-neutral agents is an effective strategy that can improve the weight profile and other metabolic outcomes. A randomized trial revealed that switching to aripiprazole led to improvements in non-HDL cholesterol levels and other metabolic parameters, suggesting that discontinuation of long-term antipsychotics had beneficial effects on eliminating metabolic abnormalities, which did not support our results [21]. A recent meta-analysis identified no significant weight changes or other metabolic changes when treatment was switched to amisulpride, paliperidone, or lurasidone [22], suggesting that the previous metabolic abnormalities remained even after switching antipsychotics, which is supported by other results. These discrepancies may be attributable to the different drugs that were switched, the switch being made directly without intervals, and the shorter duration of the original antipsychotic drug treatment. Our study further suggests that even after discontinuation of antipsychotics and recovery from metabolic abnormalities, the use of antipsychotics is still associated with more serious metabolic abnormalities in patients with previous exposure to high-metabolic-risk antipsychotics. Therefore, previous exposure to antipsychotics, especially high-metabolic-risk antipsychotics, may be a useful predictor of metabolic disturbances in schizophrenia. It is assumed that patients with a history of antipsychotics exposure may have metabolic memory related to antipsychotics with different metabolic risks, which means that when the antipsychotic drug is discontinued, the patient’s metabolism returns to normal, but when an antipsychotic drug is used again, the metabolic memory is triggered, subsequently affecting the metabolic profile of the current antipsychotic drug. However, the current study lacks additional evidence; therefore, this must be interpreted with caution.

Another unexpected finding was that this metabolic memory is affected by the previous duration of treatment. Our results revealed that previous antipsychotic drug exposure of very short duration had a slight impact on current olanzapine-induced metabolic disorders. With increasing duration of exposure to antipsychotics, differences in the effects of exposure to antipsychotics with different metabolic risks on metabolic profiles of the current treatment gradually became apparent, with higher increases and proportions of dyslipidemia observed in patients previously exposed to high-risk antipsychotics. Previous studies demonstrated that despite very short exposure to antipsychotics, antipsychotic treatment duration had a significant effect on disturbed lipid metabolism, but not on body composition [23], which contradicts our findings. In contrast, patients with higher triglyceride levels and obese patients had longer median treatment durations than patients with lower triglyceride levels and nonobese patients, which supports our study findings [24]. However, previous studies refer to current treatment with antipsychotics, while our study refers to previous treatment with antipsychotics.

Moreover, our further logistic regression analysis supported the hypothesis that a history of antipsychotics use was strongly related to current antipsychotic-induced dyslipidemia, but not weight gain, and the association between a history of antipsychotics and dyslipidemia is likely due to the exposure to high-metabolic-risk antipsychotics. Compared to patients without antipsychotics exposure, patients exposed to antipsychotics had a 1.7-times higher risk of LDL-C ≥3.37. In particular, high-metabolic-risk antipsychotic drug exposure was associated with higher metabolic risk. Investigations of the effects of previous use of antipsychotics on metabolic abnormalities induced by current antipsychotics are scarce. A meta-analysis suggested that the rates of metabolic syndrome for unmedicated patients and first-episode patients are considerably lower than that for patients with a history of antipsychotics use (35.3%), which strongly supports our results; however, unlike our study, these unmedicated patients and first-episode patients were not currently using medication [25]. Consistent with a previous study, our study revealed that sex and early changes in weight were also potential predictors of adverse metabolic reactions to antipsychotics [9].

Our study provides a novel strategy of clinical medication guidance for patients with a history of exposure to antipsychotics, especially high-metabolic-risk antipsychotics. Inadequate screening for metabolic problems is evident in real clinical practice. It was reported that only 25% and 10% of patients initiating AAPs are screened for glucose and lipid abnormalities, respectively [26]. Early monitoring of changes in blood lipid levels may help clinicians predict whether individuals will experience significant increases in metabolic parameters, thereby allowing assessment of potential risks and benefits earlier during treatment. However, this does not mean that high-metabolic-risk antipsychotics should be avoided. Many high-metabolic-risk antipsychotics, such as olanzapine and clozapine, particularly clozapine, have high clinical efficacy. Therefore, high-metabolic-risk antipsychotics can be used for short-term control of acute symptoms because the current study suggested that short-term use of high-metabolic-risk antipsychotics is not a metabolic risk factor for re-use of antipsychotics. Additionally, a previous study indicated that switching antipsychotics to agents with a lower metabolic risk can improve the weight profile and other metabolic outcomes without affecting control of psychiatric symptoms [27]. Together, these data indicate that high-metabolic-risk antipsychotics can be used to control acute symptoms during the early stage of disease, and that switching to low-metabolic-risk antipsychotics can be done after symptoms have stabilized. This may be an effective strategy for counteracting severe metabolic abnormalities during treatment with antipsychotics.

Our study had several limitations. First, other variables, such as early changes in fasting triglycerides and glucose mentioned in other literature were not available in the current study, and were not therefore included in the analysis as a control variable, which may have had an impact on the results. Additionally, we may not have strictly followed the STROBE criteria, although we tried our best to do so. Next, the exact dose of previous antipsychotics was not taken into account, and previous medication was supervised by family members, so there may have been differences in treatment compliance. Furthermore, all patients enrolled in the current study were treated with olanzapine, which is associated with high metabolic risk, and there was no control group that received low-metabolic-risk antipsychotics. Therefore, it is not known whether previous exposure to antipsychotics is an effective predictor of different metabolic risks. Additionally, we did not monitor activity levels and patient living habits during the study period. Therefore, natural between-subject differences in physical activity and diet may have influenced their energy expenditure, thereby affecting our results. We also did not explore potential mechanisms in the current study; therefore, further research is needed. The observation period during the current study was only 6 weeks. We did not continue to explore the predictive effects of the history of antipsychotic treatment on long-term metabolic risk with the current antipsychotic treatment.

## Conclusion

In conclusion, despite these limitations, our results provide preliminary evidence that a history of exposure to antipsychotics, particularly high-metabolic-risk antipsychotics, may be an effective predictor of current antipsychotic-induced metabolic disturbances. Furthermore, the association between previous antipsychotic drug treatment and metabolic risk with the current antipsychotic drug treatment is influenced by the duration of the previous treatment.

## Supplementary Information

**Additional file 1.**

**Additional file 2.**

## Data Availability

The datasets generated and/or analysed during the current study are not publicly available due to limitations of ethical approval involving the patient data and anonymity but are available from the corresponding author on reasonable request.

## Abbreviations

AAPs: Atypical antipsychotics
HDL-C: High-density lipoprotein cholesterol
BMI: Body mass index
aOR: Adjusted odds ratio
CI: Confidence interval
