# Supplementary figures and images for: Previous exposure to antipsychotic drug treatment is an effective predictor of metabolic disturbances experienced with current antipsychotic drug treatments
Source: BMC Psychiatry. 2022 Mar 21;22:210. doi: 10.1186/s12888-022-03853-y (PMC8935760; doi:10.1186/s12888-022-03853-y)

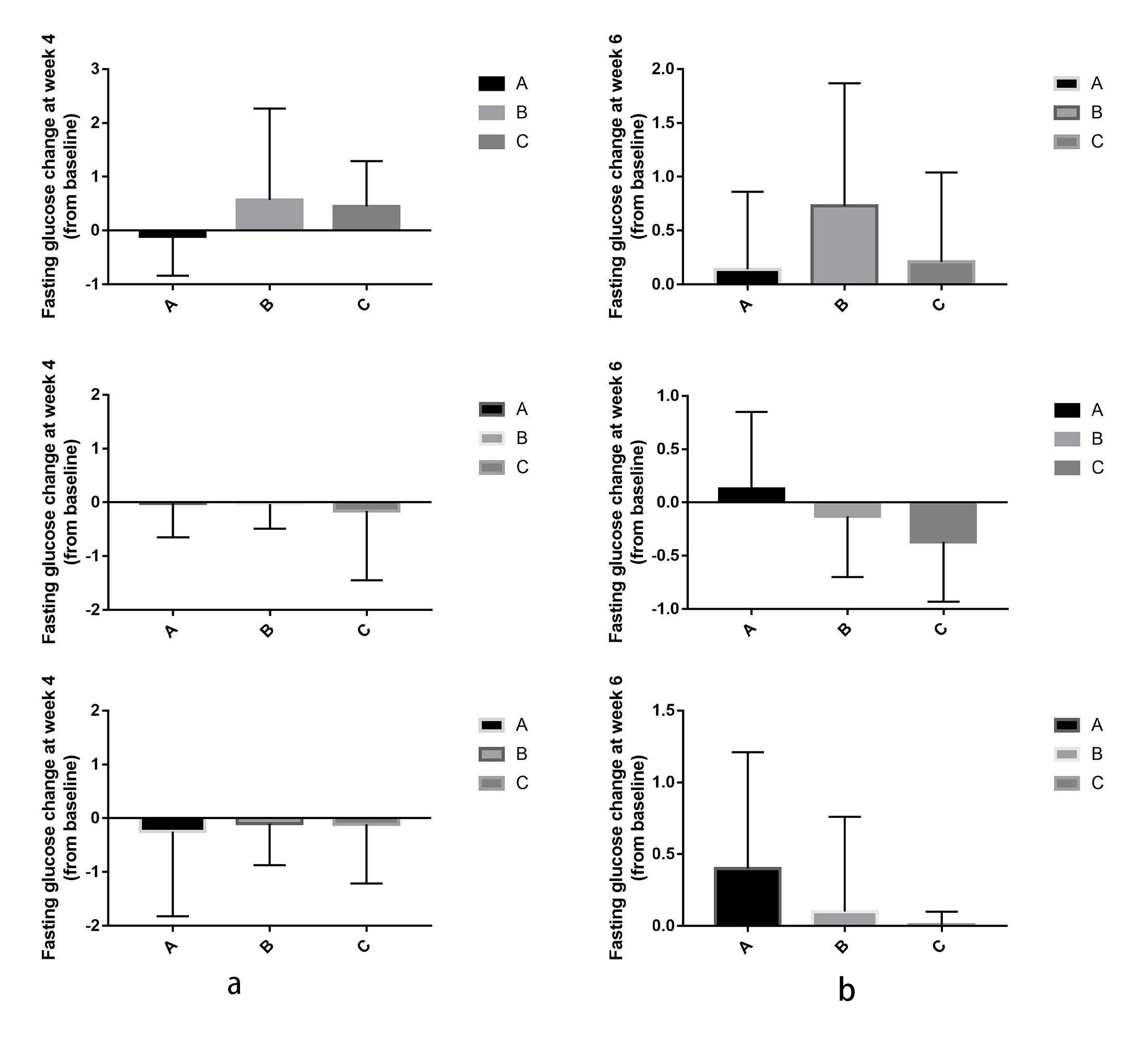

Supplement: Supplementary file 1 — Additional file 1. [file 12888_2022_3853_MOESM1_ESM.jpg]
