# Supplementary material for: Previous exposure to antipsychotic drug treatment is an effective predictor of metabolic disturbances experienced with current antipsychotic drug treatments
Source: BMC Psychiatry. 2022 Mar 21;22:210. doi: 10.1186/s12888-022-03853-y (PMC8935760; doi:10.1186/s12888-022-03853-y)
Supplement: Supplementary file 2 — Additional file 2. [file 12888_2022_3853_MOESM2_ESM.docx]

Supplementary Table1. Subgroup analysis of changes of body weight and metabolic parameters between groups during different treatment periods.

| Variable | Antipsychotic-naïve group | Previous antipsychotic-use group | *Z* | *P* | *ES* |
| --- | --- | --- | --- | --- | --- |
| ＜2year |  |  |  |  |  |
| Weight |  |  |  |  |  |
| Week4 | 2.75±3.11 | 1.70±1.92 | 3.61 | 0.136 | 0.297 |
| Week6 | 4.45±3.90 | 2.77±2.32 | 9.12 | 0.043 | 0.290 |
| BMI |  |  |  |  |  |
| Week4 | 0.88±2.31 | 0.38±0.58 | 7.56 | 0.290 | 0.292 |
| Week6 | 1.67±1.58 | 1.04±0.90 | 8.55 | 0.060 | 0.291 |
| Cholesterol |  |  |  |  |  |
| Week4 | 0.43±0.80 | 0.49±0.66 | -0.29 | 0.774 | 0.301 |
| Week6 | 0.50±0.86 | 0.56±0.75 | -0.29 | 0.771 | 0.301 |
| Triglyceride |  |  |  |  |  |
| Week4 | 0.55±1.00 | 0.44±0.95 | 0.45 | 0.656 | 0.300 |
| Week6 | 0.57±0.82 | 0.41±0.82 | 0.82 | 0.414 | 0.300 |
| HDL-C |  |  |  |  |  |
| Week4 | -0.05±0.27 | 0.08±0.29 | -1.86 | 0.068 | 0.303 |
| Week6 | -0.05±0.31 | 0.05±0.26 | -1.42 | 0.160 | 0.303 |
| LDL-C |  |  |  |  |  |
| Week4 | 0.21±0.56 | 0.40±0.70 | -1.22 | 0.228 | 0.302 |
| Week6 | 0.28±0.70 | 0.43±0.81 | -0.85 | 0.396 | 0.302 |
| Fasting glucose |  |  |  |  |  |
| Week4 | -0.11±0.73 | 0.49±1.17 | -2.49 | 0.016 | 0.304 |
| Week6 | 0.14±0.72 | 0.39±0.96 | -1.31 | 0.194 | 0.302 |
| 2-5year |  |  |  |  |  |
| Weight |  |  |  |  |  |
| Week4 | 2.19±3.89 | 1.56±1.86 | 0.84 | 0.405 | 0.300 |
| Week6 | 3.24±4.16 | 1.76±2.67 | 1.67 | 0.101 | 0.299 |
| BMI |  |  |  |  |  |
| Week4 | 0.36±1.27 | 0.19±0.41 | 0.76 | 0.453 | 0.300 |
| Week6 | 1.33±1.74 | 0.63±0.99 | 1.98 | 0.053 | 0.299 |
| Cholesterol |  |  |  |  |  |
| Week4 | 0.05±0.79 | 0.19±1.50 | -0.41 | 0.685 | 0.301 |
| Week6 | 0.23±0.74 | 0.47±0.97 | -1.03 | 0.309 | 0.302 |
| Triglyceride |  |  |  |  |  |
| Week4 | 0.68±0.97 | 0.33±0.98 | 1.34 | 0.185 | 0.299 |
| Week6 | 0.77±0.68 | 0.33±1.19 | 1.62 | 0.111 | 0.299 |
| HDL-C |  |  |  |  |  |
| Week4 | -0.18±0.47 | -0.16±0.47 | -0.07 | 0.942 | 0.301 |
| Week6 | -0.02±0.49 | 0.00±0.34 | -0.19 | 0.850 | 0.301 |
| LDL-C |  |  |  |  |  |
| Week4 | 0.12±0.67 | 0.26±0.76 | -0.75 | 0.457 | 0.302 |
| Week6 | 0.05±0.49 | 0.42±0.80 | -1.97 | 0.054 | 0.303 |
| Fasting glucose |  |  |  |  |  |
| Week4 | -0.02±0.63 | -0.10±1.00 | 0.36 | 0.722 | 0.301 |
| Week6 | 0.13±0.72 | -0.07±0.57 | 2.33 | 0.124 | 0.298 |
| ＞5year |  |  |  |  |  |
| Weight |  |  |  |  |  |
| Week4 | 3.48±3.15 | 1.57±2.84 | 3.08 | 0.003 | 0.297 |
| Week6 | 4.61±3.68 | 2.24±3.81 | 2.93 | 0.004 | 0.298 |
| BMI |  |  |  |  |  |
| Week4 | 0.86±1.31 | 0.28±0.76 | 2.91 | 0.004 | 0.298 |
| Week6 | 2.34±3.78 | 0.80±1.49 | 3.27 | 0.001 | 0.297 |
| Cholesterol |  |  |  |  |  |
| Week4 | -0.10±2.06 | 0.31±1.18 | 0.357 | 0.181 | 0.301 |
| Week6 | 0.52±1.14 | 0.24±0.94 | 0.349 | 0.207 | 0.301 |
| Triglyceride |  |  |  |  |  |
| Week4 | 0.57±1.28 | 0.74±0.92 | 0.242 | 0.448 | 0.301 |
| Week6 | 0.85±1.03 | 0.74±1.18 | 0.439 | 0.661 | 0.300 |
| HDL-C |  |  |  |  |  |
| Week4 | -0.11±0.58 | -0.03±0.39 | -0.80 | 0.425 | 0.302 |
| Week6 | 0.16±0.50 | -0.06±0.35 | 2.60 | 0.010 | 0.298 |
| LDL-C |  |  |  |  |  |
| Week4 | -0.18±1.19 | 0.28±0.87 | -2.25 | 0.026 | 0.304 |
| Week6 | 0.25±0.72 | 0.68±0.71 | -2.70 | 0.008 | 0.304 |
| Fasting glucose |  |  |  |  |  |
| Week4 | -0.25±1.57 | -0.11±0.96 | -0.57 | 0.571 | 0.302 |
| Week6 | 0.40±0.81 | 0.05±0.81 | 1.95 | 0.053 | 0.299 |

HDL-C, high-density lipoprotein cholesterol; LDL-C, low-density lipoprotein cholesterol

Supplementary Table 2. Subgroup analysis of changes of body weight and metabolic parameters among groups during different treatment periods.

| Variable | Antipsychotic-naïve group | Low-metabolic risk antipsychotics group | High-metabolic risk antipsychotics group | *H* | *P* | *ES* |
| --- | --- | --- | --- | --- | --- | --- |
| ＜2year |  |  |  |  |  |  |
| Weight |  |  |  |  |  |  |
| Week4 | 2.75±3.11 | 2.33±1.94 | 1.37±1.89 | 3.61 | 0.165 | 0.366 |
| Week6 | 4.45±3.90 | 3.90±1.93 | 1.86±2.00 | 9.12 | 0.010 | 0.361 |
| BMI |  |  |  |  |  |  |
| Week4 | 0.88±2.31 | 0.82±0.63 | 0.15±0.39 | 7.56 | 0.023 | 0.362 |
| Week6 | 1.67±1.58 | 1.70±0.80 | 0.68±0.78 | 8.55 | 0.014 | 0.361 |
| Cholesterol |  |  |  |  |  |  |
| Week4 | 0.43±0.80 | 0.44±0.46 | 0.51±0.75 | 0.52 | 0.975 | 0.369 |
| Week6 | 0.50±0.86 | 0.46±0.65 | 0.45±1.00 | 0.25 | 0.883 | 0.369 |
| Triglyceride |  |  |  |  |  |  |
| Week4 | 0.55±1.00 | 0.79±0.74 | 0.25±1.01 | 6.10 | 0.043 | 0.363 |
| Week6 | 0.57±0.82 | 0.35±0.33 | 0.45±1.00 | 1.38 | 0.501 | 0.368 |
| HDL-C |  |  |  |  |  |  |
| Week4 | -0.05±0.27 | 0.13±0.33 | 0.05±0.27 | 3.39 | 0.183 | 0.366 |
| Week6 | -0.05±0.31 | 0.05±0.23 | 0.05±0.29 | 1.15 | 0.298 | 0.368 |
| LDL-C |  |  |  |  |  |  |
| Week4 | 0.21±0.56 | 0.19±0.60 | 0.52±0.74 | 2.13 | 0.344 | 0.367 |
| Week6 | 0.28±0.70 | -0.05±0.64 | 0.69±0.78 | 5.97 | 0.051 | 0.364 |
| Fasting glucose |  |  |  |  |  |  |
| Week4 | -0.11±0.73 | 0.57±1.70 | 0.45±0.84 | 4.93 | 0.085 | 0.365 |
| Week6 | 0.14±0.72 | 0.73±1.14 | 0.21±0.83 | 2.42 | 0.298 | 0.367 |
| 2-5year |  |  |  |  |  |  |
| Weight |  |  |  |  |  |  |
| Week4 | 2.19±3.89 | 1.48±1.37 | 1.62±2.19 | 1.23 | 0.541 | 0.368 |
| Week6 | 3.24±4.16 | 1.71±2.30 | 1.80±2.30 | 7.02 | 0.030 | 0.363 |
| BMI |  |  |  |  |  |  |
| Week4 | 0.36±1.27 | 0.19±0.51 | 0.19±0.32 | 7.56 | 0.294 | 0.362 |
| Week6 | 1.33±1.74 | 0.77±1.08 | 0.53±0.94 | 8.97 | 0.011 | 0.361 |
| Cholesterol |  |  |  |  |  |  |
| Week4 | 0.05±0.79 | -0.22±1.38 | 0.50±1.54 | 5.16 | 0.076 | 0.364 |
| Week6 | 0.23±0.74 | -0.11±0.59 | 0.91±0.96 | 10.86 | 0.004 | 0.359 |
| Triglyceride |  |  |  |  |  |  |
| Week4 | 0.68±0.97 | 0.10±0.64 | 0.51±1.16 | 3.17 | 0.205 | 0.366 |
| Week6 | 0.77±0.68 | 0.39±1.30 | 0.82±1.14 | 6.15 | 0.644 | 0.363 |
| HDL-C |  |  |  |  |  |  |
| Week4 | -0.18±0.47 | -0.13±0.56 | -0.18±1.13 | 1.63 | 0.443 | 0.368 |
| Week6 | -0.02±0.49 | -0.08±0.29 | 0.06±0.37 | 1.00 | 0.607 | 0.368 |
| LDL-C |  |  |  |  |  |  |
| Week4 | 0.12±0.67 | -0.04±0.72 | 0.49±0.71 | 6.00 | 0.050 | 0.364 |
| Week6 | 0.05±0.49 | -0.16±0.43 | 0.84±0.75 | 17.66 | ＜0.001 | 0.352 |
| Fasting glucose |  |  |  |  |  |  |
| Week4 | -0.02±0.63 | -0.03±0.46 | -0.16±1.29 | 0.01 | 0.994 | 0.369 |
| Week6 | 0.13±0.72 | -0.13±0.57 | -0.37±0.56 | 5.81 | 0.055 | 0.364 |
| ＞5year |  |  |  |  |  |  |
| Weight |  |  |  |  |  |  |
| Week4 | 3.48±3.15 | 2.97±1.89 | 0.49±2.99 | 1.23 | ＜0.001 | 0.368 |
| Week6 | 4.61±3.68 | 3.98±1.78 | 0.91±4.40 | 7.02 | ＜0.001 | 0.363 |
| BMI |  |  |  |  |  |  |
| Week4 | 0.86±1.31 | 0.59±0.49 | 0.05±0.85 | 7.56 | ＜0.001 | 0.362 |
| Week6 | 2.34±3.78 | 1.42±0.73 | 0.33±1.59 | 8.97 | ＜0.001 | 0.361 |
| Cholesterol |  |  |  |  |  |  |
| Week4 | -0.10±2.06 | 0.55±1.14 | 1.13±1.19 | 5.16 | 0.040 | 0.364 |
| Week6 | 0.52±1.14 | 0.30±0.91 | 0.20±0.97 | 10.86 | 0.108 | 0.359 |
| Triglyceride |  |  |  |  |  |  |
| Week4 | 0.57±1.28 | 0.92±0.96 | 0.59±0.87 | 3.17 | 0.146 | 0.366 |
| Week6 | 0.85±1.03 | 0.75±1.12 | 0.73±1.29 | 6.15 | 0.596 | 0.363 |
| HDL-C |  |  |  |  |  |  |
| Week4 | -0.11±0.58 | 0.02±0.40 | -0.08±0.38 | 1.63 | 0.094 | 0.368 |
| Week6 | 0.16±0.50 | 0.00±0.37 | -0.11±0.32 | 1.00 | 0.034 | 0.368 |
| LDL-C |  |  |  |  |  |  |
| Week4 | -0.18±1.19 | 0.44±0.89 | 0.17±0.84 | 6.00 | 0.024 | 0.364 |
| Week6 | 0.25±0.72 | 0.49±0.62 | 0.82±0.74 | 17.66 | 0.012 | 0.352 |
| Fasting glucose |  |  |  |  |  |  |
| Week4 | -0.25±1.57 | -0.10±0.77 | -0.11±1.10 | 1.85 | 0.397 | 0.367 |
| Week6 | 0.40±0.81 | 0.10±0.66 | 0.01±0.09 | 5.44 | 0.066 | 0.364 |

HDL-C, high-density lipoprotein cholesterol; LDL-C, low-density lipoprotein cholesterol

Supplementary Table3. Dyslipidemia defined by each single outcome measurement in antipsychotic-naïve group, Low-metabolic risk antipsychotics group and High-metabolic risk antipsychotics group during different treatment periods.

|  | Antipsychotic-naïve group | | Low-metabolic risk antipsychotics group | | High-metabolic risk antipsychotics group. | | *Chi-Sq* | *P-value* | *ES* |
| --- | --- | --- | --- | --- | --- | --- | --- | --- | --- |
|  | *N* | *%* | *N* | *%* | *N* | *%* |  |  |  |
| ＜2year |  |  |  |  |  |  |  |  |  |
| Total cholesterol (≥5.18mmol l–1) | 11 | 17.7 | 1 | 11.1 | 6 | 35.3 | 3.06 | 0.216 | 0.183 |
| Triglyceride (≥1.70 mmol l–1) | 21 | 33.9 | 2 | 22.2 | 7 | 41.2 | 0.95 | 0.677 | 0.103 |
| HDL-C (<1.04 mmol l–1) | 12 | 19.3 | 1 | 11.1 | 3 | 17.6 | 0.36 | 0.914 | 0.164 |
| LDL-C (≥3.37 mmol l–1) | 6 | 9.7 | 1 | 11.1 | 2 | 11.7 | 0.07 | 0.965 | 0.129 |
| 2-5year |  |  |  |  |  |  |  |  |  |
| Total cholesterol (≥5.18mmol l–1) | 7 | 29.2 | 2 | 14.3 | 7 | 36.8 | 6.38 | 0.041 | 0.317 |
| Triglyceride (≥1.70 mmol l–1) | 14 | 51.9 | 20 | 48.7 | 20 | 40.0 | 1.52 | 0.303 | 0.201 |
| HDL-C (<1.04 mmol l–1) | 3 | 12.5 | 4 | 30.8 | 6 | 33.3 | 2.95 | 0.228 | 0.226 |
| LDL-C (≥3.37 mmol l–1) | 2 | 8.3 | 1 | 7.7 | 9 | 50.0 | 12.46 | 0.002 | 0.430 |
| ＞5year |  |  |  |  |  |  |  |  |  |
| Total cholesterol (≥5.18mmol l–1) | 9 | 33.3 | 12 | 30.8 | 11 | 22 | 1.43 | 0.490 | 0.110 |
| Triglyceride (≥1.70 mmol l–1) | 7 | 41.2 | 8 | 42.1 | 20 | 40 | 1.518 | 0.468 | 0.114 |
| HDL-C (<1.04 mmol l–1) | 5 | 18.5 | 4 | 10.3 | 17 | 40.5 | 7.75 | 0.021 | 0.251 |
| LDL-C (≥3.37 mmol l–1) | 6 | 22.2 | 8 | 20.5 | 8 | 16.3 | 0.46 | 0.793 | 0.063 |

HDL-C, high-density lipoprotein cholesterol; LDL-C, low-density lipoprotein cholesterol

Supplementary Table4. Weight gain more than 7% of initial weight in antipsychotic-naïve group, Low-metabolic risk antipsychotics group and High-metabolic risk antipsychotics group during different treatment periods.

| Weight gain more than 7% of initial weight | Antipsychotic-naïve group | | Low-metabolic risk antipsychotics group | | High-metabolic risk antipsychotics group | | *Chi-Sq* | *P-value* | *ES* |
| --- | --- | --- | --- | --- | --- | --- | --- | --- | --- |
|  | N | % | N | % | N | % |  |  |  |
| ＜2year | 25 | 40.3 | 6 | 66.7 | 10 | 58.8 | 3.46 | 0.177 | 0.194 |
| 2-5year | 10 | 41.7 | 9 | 60 | 3 | 15 | 7.75 | 0.022 | 0.341 |
| ＞5year | 4 | 13.8 | 13 | 33.3 | 3 | 5.9 | 12.16 | 0.002 | 0.304 |

HDL-C, high-density lipoprotein cholesterol; LDL-C, low-density lipoprotein cholesterol

Supplementary Table5. Univariate conditional logistic regression analyses for the risk of metabolic adverse reactions.

| Variable | weight gain exceeding 7% | | cholesterol ≥5.18 mmol/L^–1^ | | triglycerides ≥1.70 mmol/L^˗1^ | | HDL-C <1.04 mmol/L^–1^ | | LDL-C ≥3.37 mmol/L^–1^ | |
| --- | --- | --- | --- | --- | --- | --- | --- | --- | --- | --- |
|  | Adjusted OR (95% CI) | P | Adjusted OR (95% CI) | P | Adjusted OR (95% CI) | P | Adjusted OR (95% CI) | P | Adjusted OR (95% CI) | P |
| age | 0.973(0.941-1.006) | 0.105 | 1.052(1.015-1.090) | 0.006 | 1.030(0.998-1.062) | 0.064 | 1.031(0.993-1.070) | 0.107 | 1.038(0.996-1.081) | 0.075 |
| sex | 1.285(0.763-2.163) | 0.046 | 0.741(0.421-1.305) | 0.299 | 0.661(0.403-1.084) | 0.101 | 2.581(1.378-3.834) | 0.003 | 2.187(1.106-3.326) | 0.025 |
| baseline weight | 0.962(00.938-0.988) | 0.004 | 1.018(0.993-1.045) | 0.163 | 1.030(1.007-1.054) | 0.012 | 1.038(1.010-1.066) | 0.008 | 1.018(0.989-1.048) | 0.233 |
| early changes in weight | 1.340(1.137-1.579) | <0.001 | 0.958(0.857-1.133) | 0.834 | 0.895(0.873-1.111) | 0.804 | 0.908(0.782-1.054) | 0.203 | 0.854(0.721-1.012) | 0.069 |
| antipsychotics exposure | 0.801(0.476-1.350) | 0.405 | 1.062(0.600-1.878) | 0.837 | 0.949(0.578-1.560) | 0.838 | 1.480(0.800-2.736) | 0.212 | 1.768(1.005-3.531) | 0.017 |
| low-metabolic-risk antipsychotics | 1.559(0.831-2.925) | 0.167 | 0.845(0.400-1.787) | 0.659 | 0.978(0.522-1.831) | 0.945 | 0.805(0.342-1.896) | 0.619 | 1.387(0.576-3.340) | 0.466 |
| high-metabolic-risk antipsychotics | 0.433(0.223-0.842) | 0.104 | 1.233(0.650-2.337) | 0.521 | 0.929(0.526-1.642) | 0.801 | 2.084(1.068-4.069) | 0.031 | 2.067(1.069-4.441) | 0.016 |

Supplementary Figure 1. Subgroup analysis of changes in fasting glucose among different groups after 4 weeks and 6 weeks of olanzapine treatment. (a) Changes in fasting glucose among different groups after 4 weeks of olanzapine treatment. (b) Changes in fasting glucose among different groups after 6 weeks of olanzapine treatment. The top graph indicates changes in fasting glucose among different groups with 1 to 2 years of previous antipsychotics exposure. The middle graph represents changes in body weight and metabolic parameters among different groups with 2 to 5 years of previous antipsychotics exposure. The bottom graph represents changes in body weight and metabolic parameters among different groups with more than 5 years of previous antipsychotics exposure. A, B, and C represent the antipsychotic-naïve group, low-metabolic-risk antipsychotics group, and high-metabolic-risk antipsychotics group, respectively.
